# EEG-Based Mental Tasks Recognition via a Deep Learning-Driven Anomaly Detector

**DOI:** 10.3390/diagnostics12122984

**Published:** 2022-11-29

**Authors:** Abdelkader Dairi, Nabil Zerrouki, Fouzi Harrou, Ying Sun

**Affiliations:** 1 Computer Science Department, University of Science and Technology of Oran-Mohamed Boudiaf (USTO-MB), El Mnaouar, BP 1505, Bir El Djir 31000, Algeria; 2 Design and Implementation of Intelligent Machines (DIIM) Team, Center for Development of Advanced Technologies, Baba Hassen 16081, Algeria; 3 Computer, Electrical and Mathematical Sciences and Engineering (CEMSE) Division, King Abdullah University of Science and Technology (KAUST), Thuwal 23955-6900, Saudi Arabia

**Keywords:** deep learning, motor imagery, Isolation Forest, anomaly detection, EEG signals classification

## Abstract

This paper introduces an unsupervised deep learning-driven scheme for mental tasks’ recognition using EEG signals. To this end, the Multichannel Wiener filter was first applied to EEG signals as an artifact removal algorithm to achieve robust recognition. Then, a quadratic time-frequency distribution (QTFD) was applied to extract effective time-frequency signal representation of the EEG signals and catch the EEG signals’ spectral variations over time to improve the recognition of mental tasks. The QTFD time-frequency features are employed as input for the proposed deep belief network (DBN)-driven Isolation Forest (iF) scheme to classify the EEG signals. Indeed, a single DBN-based iF detector is constructed based on each class’s training data, with the class’s samples as inliers and all other samples as anomalies (i.e., one-vs.-rest). The DBN is considered to learn pertinent information without assumptions on the data distribution, and the iF scheme is used for data discrimination. This approach is assessed using experimental data comprising five mental tasks from a publicly available database from the Graz University of Technology. Compared to the DBN-based Elliptical Envelope, Local Outlier Factor, and state-of-the-art EEG-based classification methods, the proposed DBN-based iF detector offers superior discrimination performance of mental tasks.

## 1. Introduction

The brain–computer interface (BCI) is a recent manner of communication where it allows translating oscillatory Electro Encephalogram (EEG) patterns into action [1]. BCI technology has proven very successful in the scientific community, since it allows the command of several devices, such as a computer, from the assisted control of the human brain [2]. It is based on the electrical brain activities of disabled patients who have lost their mobility autonomy [3]. In other words, several motivations can be highlighted for designing an MI-based system. Suppose one considers only the case of motor-impaired people. In that case, MI-based task recognition can solve several daily problems, such as a text-entry system, making a phone call, and wheel-chair control, especially if the recognition system has certain reliability and robustness in real time. In a more advanced stage, MI-based systems for BCI applications can be much more important if communication between two or more patients can be established via the MI-based system. It is worth noting that many researchers and engineers have developed several methods and systems for EEG signals classification during the last few years [4,5]. However, there are still several unsolved problems in motor imagery (MI)-based tasks recognition [6]. Essentially, transforming thoughts into actions via BCI remains challenging because recorded EEG signals are highly affected by background noise and different artifacts, including eye blinking, cardiac activity, and the state of stress of the patient.

Various approaches have been proposed for MI tasks classification in the last few years. For instance, the authors in [3] applied tunable-Q wavelet transform (TQWT) on EEG signals for extracting time-frequency features, and the Least-squares Support Vector Machine (LSSVM) algorithm was applied for separating between right-hand and right-foot MI tasks. In another work, the same research team subsequently [6] proposed the use of Analytic Intrinsic Mode Functions (AIMFs) based on Empirical Mode Decomposition (EMD) and Hilbert transform for the EEG signal feature extraction module. To this end, several attributes have been generated, such as peak value and spectral moment of power spectral density, and the raw moment of the first derivative of instantaneous frequency. These features are then considered inputs to the LSSVM classifier with radial basis function as kernel function, where an overall accuracy of 97.56% was obtained. The study in [7] proposed optimal allocation features to discriminate the operative information from EEG data with a minimum possible rate of variability. The classification stage was performed using Naive Bayes and LS-SVM algorithms, obtaining an accurate detection percentage of 96.36% and 96.62%, respectively. The authors in [8] presented an EEG de-noising phase using a non-linear filter based on the Multi-Scale Principal Component Analysis (MSPCA) technique. Diverse attributes were generated by the application of Empirical Mode Decomposition (EMD), discrete wavelet transform (DWT), and wavelet packet decomposition (WPD). In the classification stage, a k-nearest neighbor algorithm was applied to separate between two classes, namely right hand and foot, where a correct recognition rate of 92.8% was obtained according to their experimental results. Authors in [9] designed sliding window techniques to improve the binary classification of motor imagery, where features are extracted via Common spatial pattern (CSP), and the classification is conducted using linear discriminant analysis (LDA). They have used the BCI Competition IV-2a data dataset, which is publicly available, and demonstrated satisfactory classification performance of this approach by reaching an accuracy of around 80%. In [10], a combined approach merging an improved group least absolute shrinkage and selection operator (LASSO) is proposed for EEG signals spatial smoothing, features selection, and classification. It has been demonstrated that this approach can enhance the performance of BCI systems. In [11], a combination of cross-correlation and discrete wavelet transform (DWT) has been employed in feature generation and selection procedures to classify MI-based EEG signals. After that, the classification task was conducted by the application of five different methods, namely: multilayer perceptron neural network (MLP), probabilistic neural network (PNN), logistic regression (LR), kernelized logistic regression (KLR), and LS-SVM. In [12], an approach based on machine learning and feature selection techniques is considered for motor imagery EEG signal classification on the internet of medical things environment. To this end, actual feature sets are obtained from BCI Competition-II Dataset-III motor-imagery EEG signal using the Adaptive Auto-regressive approach. Then, an innovative fuzzified extension of the discernibility matrix is employed for feature selection. Results demonstrated that when used with SVM and Ensemble variants of classifiers, this feature selection procedure outperformed other commonly used approaches.

In recent years, there has been a growing interest in designing efficient techniques for EEG-based classification of motor imagery tasks exploiting machine learning and deep learning models. However, most of these techniques are developed in a supervised learning procedure where labeling information is needed. Essentially, this work focuses on developing a flexible and unsupervised data-driven approach to effectively identify mental tasks based on EEG signals. Here, we addressed the multiclass mental tasks classification as an anomaly detection problem employing an unsupervised deep learning model. Overall, the contributions of this study are recapitulated as follows.

- This work presents a deep learning-based anomaly detection strategy to enhance mental tasks recognition by EEG data. This strategy comprises several stages, i.e., artifacts removal, extraction of time-frequency features of EEG signals, anomaly detection, and classes discrimination. Specifically, the EEG signals are first filtered using the Multichannel Wiener filter (MWF) to remove artifacts and achieve robust recognition. We adopted a quadratic time-frequency distribution (QTFD) for extracting high-resolution time-frequency signal representation of the EEG signals. The employment of a QTFD technique is expected to improve the recognition of mental tasks by capturing the EEG signals’ spectral variations over time. The extracted time-frequency features are inputs to the proposed unsupervised deep learning-based approach for classifying the EEG signals. Indeed, this study treated multiclass classification as a multiple-binary discrimination problem. Specifically, this approach combines the desirable characteristics of both a deep belief network (DBN) and an isolation forest (IF) technique for separating mental tasks based on the time-frequency features of EEG signals. Crucially, this technique profits from the greedy learning characteristics of the DBN for extracting pertinent information from the QTFD features and the capacity of the IF detector to sense outliers. The IF algorithm’s key characteristic is its ability to reveal anomalies without using distance or density metrics. This enables eliminating computational costs related to distance computation in all distance-driven and density-driven models. In addition, the iF detector can handle large-sized datasets with many irrelevant features [13]. Indeed, a single DBN-based IF detector is constructed based on training data in the targeted class, i.e., the samples in such class are considered inliers, and all remaining samples are considered anomalies (i.e., one-vs.-rest). We evaluated the efficacy of this technique through experimental data comprising five mental tasks: mental word association, mental subtraction, spatial navigation, right-hand motor imagery, and feet motor imagery, from a publicly available database from the Institute for Knowledge Discovery, Graz University of Technology, Austria. Thus, to separate the five mental tasks by EEG signals, by using one-vs.-rest method, we constructed five DBN-IF detectors.
- Furthermore, the discrimination capabilities of the DBN-IF scheme have been compared with those of DBN-based Local Outlier Factor (LOF) and Elliptical Envelope (EE) anomaly detection methods. As we know, DBN-based LOF and EE methods have not previously been used for EEG-based mental tasks identification. The essence of LOF is based on the idea of local anomalies [14], while the EE senses anomalies by fitting an ellipse around the data utilizing the Minimum Covariance Determinant [15]. We assessed the performance of the investigated technique using four commonly used statistical scores. Results revealed that the proposed DBN-IF approach dominates the other investigated approaches.
- In addition, the results of the DBN-IF approach are compared with the state-of-the-art techniques; the results demonstrated the proposed approach’s outperformance in improving the separation of metal tasks based on EEG signals.

The remainder of this paper is organized as follows. Section 3 briefly describes the preliminary materials, including the MWF artifact removal technique, the QTFD features extractor, the DBN model, and the iF anomaly detector. Section 3 presents the proposed approach to distinguish between the five mental tasks. In Section 4, we present the used data and the obtained results. Finally, we offer conclusions in Section 5.

## 2. Related Works

Mental tasks recognition based on EEG signals is a challenging problem in EEG signal processing and analysis. Recently, deep learning models are employed to enhance medical applications in academia and industry due to their ability of extracting pertinent features of high dimensional data [16]. They demonstrated promising performance in various applications, including COVID-19 infection detection [17], Parkinson’s disease detection [18]. Various studies have investigated deep learning techniques for EEG classification problems in recent years. For instance, in [19], Bashivan et al. proposed a new representation of EEG classification problematic, where raw EEG signals are transformed into a sequence of topology multi-spectral images or frames. Inspired by the representation of images and videos classification using deep learning techniques, the authors applied a deep recurrent convolutional network (CNN) for classifying the transformed images. However, it remains challenging to preserve EEG signals’ spatial, spectral, and temporal structure after transforming them into 2D images. The study in [20] applied Extreme Learning Machine (ELM) to discriminate five mental tasks based on EEG signals. Results demonstrated that ELM obtained similar classification performance in terms of accuracy as SVMs and Backpropagation Neural Network (BPNN) classifiers. However, it has less training time compared to SVMs and BPNN. Furthermore, it has been demonstrated that smoothing the classifiers’ outputs enhances their discrimination accuracies. In [21], two approaches based on deep convolutional neural networks and deep residual learning are applied for the EEG classification of driver mental states. Data from a driving simulation platform is used to verify the effectiveness of these classifiers. Results based on intra- and inter-subject demonstrated that the two models achieved good classification performance and outperformed the LSTM- and SVM-driven classifiers. However, this study focused only on a binary classification to predict driver fatigue. In kuremoto2019mental, Kuremoto et al. proposed hybrid machine learning methods for EEG-based mental task recognition by combining SVM and neural networks (e.g., MLP, CNN, and stacked auto-encoder (SAE)), as well as the mixed SAE+MLP, CNN+MLP models. Results revealed the superior classification accuracy of the hybrid models than the traditional methods (i.e., MLP, SVM, and CNN). However, the inputs considered by these models consist of the raw EEG signals; other input alternatives can be considered for improvements, such as the preprocessed data by wavelet transform or Fourier transform. In [22], Opalka et al. adopted a Multi-Channel Convolutional Neural Networks architecture for EEG mental tasks classification. Results based on data from V from BCI Competition III demonstrated the superior classification performance of this approach with an accuracy of around 70%, surpassing alternative methods (i.e., AlexNet, VGG-16, and Cecotti’s multi-channel NN).

In [23], time-frequency features and location information are first extracted from MI EEG signals, and the short-time Fourier transform (STFT) method was then applied to proceed into a 2D image representation. In the classification phase, CNN with only 1D convolutional and one max-pooling layer was combined with stacked autoencoders (SAE), obtaining a correct recognition rate of 90.0%. In [24], a classification framework using a long short-term memory (LSTM) with one dimension-aggregate approximation as a feature extractor was proposed to classify EEG motor imagery tasks. This approach employs a softmax layer for predicting the probability of every class. Classification results based on public BCI competition data demonstrated that the LSTM-based approach outperformed the state-of-the-arts approaches using no other deep networks by obtaining an averaged accuracy of 75.28%. In [25], a hybrid approach called frequential deep belief network (FDBN) is designed to deal with Motor imagery classification problems by combining a fast Fourier transform (FFT), and wavelet package decomposition (WPD) are combined with a deep belief neural network (DBN). At first, FFT and WPD are applied to obtain frequency domain representations of EEG signals, and their output features are used to train the DBN model. Here, a softmax layer is adopted to perform the classification task. In [26], a hierarchical flow convolutional neural network (HF-CNN) model is introduced to classify forearm movements using electroencephalogram (EEG) signals. This approach has been assessed using experimental and BNCI Horizon 2020 datasets and demonstrated a moderate classification performance. This study helps develop a brain-controlled robotic arm system to perform high-level tasks. Authors in [27] proposed a Graph-based CNN network combined with an attention model for motor imagery classification. Specifically, the positioning information of EEG nodes is first represented by a graph structure, and then the CNN with attention is applied to learn EEG features. They showed that the EEG graph with more nodes significantly enhances the overall performance. Authors in [28] present a motor imagery tasks’ EEG signals classification using CNN in the brain–BCI system. Essentially, the CNN model is employed to classify the right hand and right foot MI-task using EEG signals. To this end, the CNN moded is trained with transformed EEG signals into images via time-frequency approaches, namely short-time Fourier transform (STFT) and continuous wavelet transform (CWT). Results revealed that the classification performance achieved by using CWT was significantly better than that obtained via the STFT approach. In another study [29], a classification of hand movements framework based on EEG signals is introduced using a deep attention-based LSTM network. Importantly, the attention-based LSTM is trained using time and frequency domain features extracted from the EEG signals. However, this approach is based on the use of hand-crafted features.

## 3. Materials and Methods

**Figure 1:**
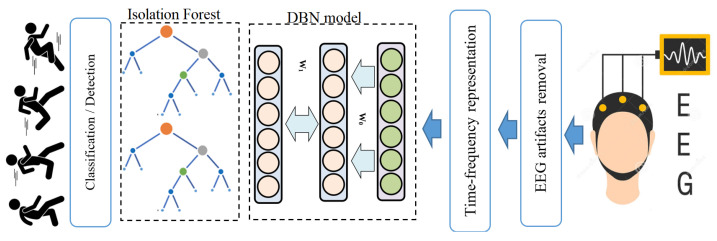
Schematic representation of the general deep learning framework for EEG-based metal tasks discrimination.

The general framework of the proposed EEG-based mental tasks recognition is schematically illustrated in Figure 1. This framework consists of five main steps: data acquisition, artifacts removal through MWF, time-frequency representation of the EEG signals using QTFD, feature extraction via the DBN model, and mental tasks recognition based on the IF anomaly detection scheme.

### 3.1. EEG Artifacts Removal Using Multi-Channel Wienner Filter

The collected EEG signals are usually tinted with glitches or spikes due to sudden changes in skin-electrode contact impedance. Specifically, this situation is often produced by the movement of the subject head, resulting in the shift of electrodes around. This movement artifact presents a very large magnitude peak that impacts a single channel or a few adjacent channels. The morphology of this kind of artifacts (with focused spatial and sparse temporal structure appearing only once in a few channels in the recording) can differ significantly compared to eye blinks artifacts, which are redundant for a subject.

**Figure 2:**
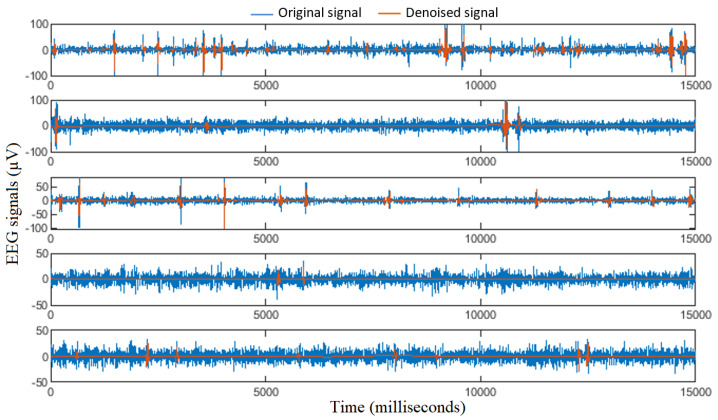
Illustration of EEG signals before and after artifacts’ removal.

This study gives EEG signals input to an MWF algorithm for EEG artifacts’ removal. The MWF algorithm is known for its efficiency using both hybrid and actual EEG data, since it can eliminate a wide range of artifacts with more satisfactory performance than current existing techniques [30]. Notably, the main idea behind MWF is that a low-rank approximation replaces the artifact covariance matrix via the generalized eigenvalue decomposition [30]. The employment of MWF as an artifact removal technique was strongly motivated by the fact that the MWF is not limited to a specific kind of artifact, and it is robust and generic for various types of EEG artifacts (other artifacts than eye blink or muscle artifacts such as movement artifact) [30]. Figure 2 provides an illustration of some examples of EEG signals before and after artifacts’ removal using the MWF algorithm.

### 3.2. Time-Frequency Representation of EEG Data via a QTFD

After obtaining relevant EEG signals by removing artifacts, they are used as input to time-frequency features through quadratic time-frequency distributions (QTFDs). This study uses QTFD to extract time-frequency components due to its capacity to catch the EEG signals’ spectral variations over time. In particular, the EEG signals could be represented using a QTFD by discriminant features that could improve the recognition ratio of distinct emotional classes. Moreover, the QTFD has been efficiently implemented to characterize EEG signals in several fields, including decoding motor imagery tasks [31]. The main idea behind using QTFD components is to consider the nonlinearity aspect in mapping EEG signals; moreover, QTFDs are invariant to the time-frequency shift, which can avoid some limitations encountered using other time-frequency analysis techniques, such as Scale-invariant feature transform or Wavelet Transform [31]. In order to calculate the QTFD components of EEG signals, a sliding window is used to split the EEG signal of each channel into a set of EEG segments.

For each EEG segment, the QTFD-based time-frequency representations are based on the Hilbert transform and Wigner–Ville distribution [32]. Given the time evolution EEG segment (real-valued signal) s(t), the QTFD components are computed, as follows:

(1) a(t)=s(t)+jHT(s(t)),

where a(t) and HT represent the Hilbert transform and the analytic signal, respectively. The Wigner–Ville distribution (WVD) of the signal a(t) is then computed as follows:

(2) $$WVD_{a}\left(t,f\right)=\int_{-\infty }^{\infty }a\left(t+\frac{\tau }{2}\right)a^{*}\left(t-\frac{\tau }{2}\right)e^{-j2\pi \tau f}\partial \tau ,$$

where τ and $a^{*}\left(t\right)$ represent the parameter of the centered formulation and the complex conjugate of a(t), respectively. Finally, to obtain QTFD, we simply convolve the computed $WVD_{a}\left(t,f\right)$ with a time-frequency kernel K(t,f).

(3) $$\varrho_{a}\left(t,f\right)=\int_{-\infty }^{\infty }\int_{-\infty }^{\infty }WVD_{a}\left(\phi ,\tau \right)K\left(\phi ,\tau \right)e^{-j2\pi f\tau -j2\pi t\phi }\partial \tau \partial \phi ,$$

where $\varrho_{a}\left(t,f\right)$ represents the QTFD of a(t). In the recognition cases, exponential time-frequency kernel is generally used to moderate the aspect of the cross-terms and keep a satisfactory resolution in both the time- and frequency-domain. In the present case, the exponential kernel is expressed as:

(4) $$K\left(t,f\right)=\exp\left(-\frac{t^{2}f^{2}}{\alpha^{2}}\right).$$

**Figure 3:**
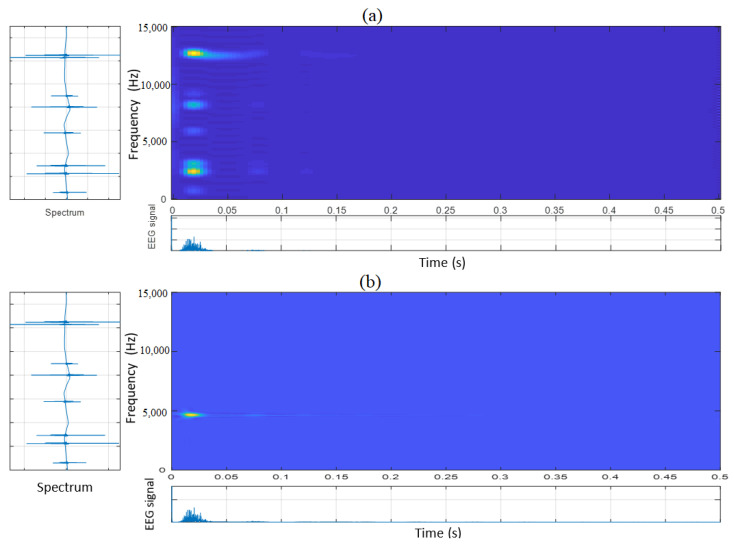
A practical example of Choi-Williams decomposition (QTFD) of EEG signal.

Here, α represents the parameter controlling the suppression of the cross-terms. In other words, the exponential kernel tends to eliminate the interference, which is away from the origin. The QTFD based on the exponential kernel is called the Choi-Williams distribution (CWD). More details and explanations about the QTFD or CWD extraction process can be found in [31]. To be more explicit and provide more details concerning QTFD feature extraction, Figure 3a,b illustrates two examples of the Choi-Williams decomposition. For instance, in Figure 3a, the examined EEG signal is given in the bottom panel, the response in the frequency domain is shown in the right panel, and its corresponding spectrum is displayed in the left panel.

The extracted QTFD-based time-frequency features will be used as input by the proposed deep learning-based anomaly detection approach to discriminate between the various mental tasks.

### 3.3. Deep Belief Network (DBN)

**Figure 4:**
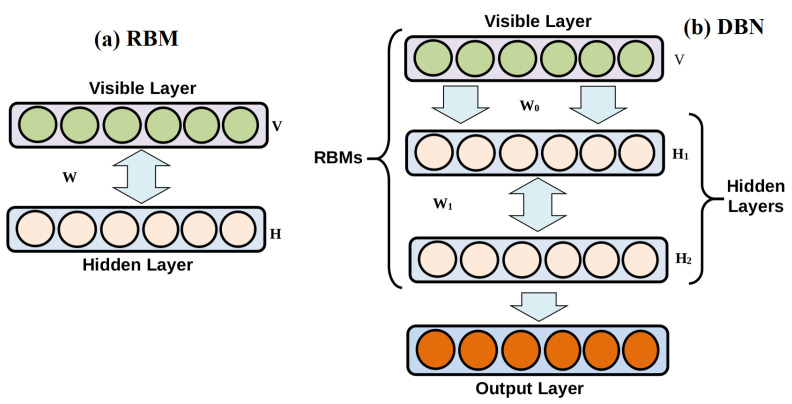
Schematic illustration of (**a**) RBM and (**b**) DBN models.

DBNs are probabilistic generative models designed by stacked restricted Boltzmann machines (RBMs). RBMs are a powerful tool for extracting and representing data adopted in machine learning [33] (see Figure 4a). As schematized in Figure 4a, RBM is a variant of the conventional Boltzmann Machine (BM), which removes all connections in the same layer, and only the connections between visible and hidden layers are preserved [33].

RBMs are energy-driven models and were employed as generative models for different kinds of data [34], including text, speech, and images. The energy of join structure is expressed [35]:

(5) $$Energy\left(v,h\right)=-\sum_{i=1}^{m}\sum_{j=1}^{n}W_{ij}v_{i}h_{j},-\sum_{i=1}^{m}b_{i}v_{i}-\sum_{j=1}^{n}c_{j}h_{j},$$

where $W_{ij}$ represents the element of *W* which connects the the *i*th visible variable $v_{i}$ to the *j*th hidden variable $h_{j}$, *b* and *c* denote the parameters of the model.

Then, the underlying Boltzmann distribution can be computed as [36]:

(6) $$\begin{array}{c} P\left(v,h\right)=\frac{\exp(-Energy(v,h))}{\sum_{v}\sum_{h}\exp\left(-Energy\left(v,h\right)\right)}=\frac{\prod_{ij}e^{W_{ij}v_{i}h_{j}}\prod_{i}e^{b_{i}v_{i}}\prod_{j}e^{a_{j}h_{j}}}{\sum_{v}\sum_{h}\exp\left(-Energy\left(v,h\right)\right)}, \end{array}$$

Since only *v* is observed, the hidden variables *h* are marginalized.

(7) $$P\left(v\right)=\sum_{h}\frac{e^{-Energy(v,h)}}{\sum_{v}\sum_{h}\exp\left(-Energy\left(v,h\right)\right)},$$

where P(v) refers to the probability allocated by the mode to a visible vector *v*. As there is no connection between the nodes at the same layer (Since the intra-connections are absent at both layers), the corresponding conditional probabilities are:

(8) $$P\left(v|h\right)=\prod_{i}p\left(v_{i}|h\right),\mathrm{and}\;\;P\left(h|v\right)=\prod_{j}p\left(h_{j}|v\right).$$

For binary data, equations in (8) can be reformulated as:

(9) $$P\left(v_{i}=1|h\right)=\sigma \left(\sum_{j}W_{ij}h_{j}+c_{i}\right),$$

(10) $$P\left(h_{j}=1|v\right)=\sigma \left(\sum_{i}W_{ij}v_{i}+b_{j}\right),$$

where σ(.) denotes the logistic function and $\sigma \left(x\right)={\left(1+\exp\left(-x\right)\right)}^{-1}$.

DBNs are built up by stacking RBMs (Figure 4b) and trained in an unsupervised manner for extracting pertinent features from the input data. They proved to be effective in uncovering layer-by-layer complex nonlinearity. In [35], a fast learning strategy for DBN was introduced, where the joint distribution between observed vector *x* and *ℓ* hidden layers $h^{k}$ is obtained as [34],

(11) $$P\left(x,h^{1},\cdots ,h^{l}\right)=\left(\prod_{k=0}^{\ell -2}P\left(h^{k}|h^{k+1}\right)\right)P\left(h^{\ell -1},h^{\ell }\right),$$

where $x=h^{0}$ and $P(h^{k}|h^{k+1})$ is a visible given hidden conditional distribution in an RBM associated with level *k* of the DBN, and $P(h^{\ell -1},h^{\ell })$ is the joint distribution in the top-level RBM.

Basically, including more layers in the DBN enhances modeling power. The accuracy of the energy expression could be improved by incorporating more additional layers into the DBN model [34]. However, little is earned by employing more than three hidden layers in practice. For instance, in this study, we stacked two RBMs (Figure 4b) to construct our DBN model without any labeling information.

### 3.4. Isolation Forest Approach

**Figure 5:**
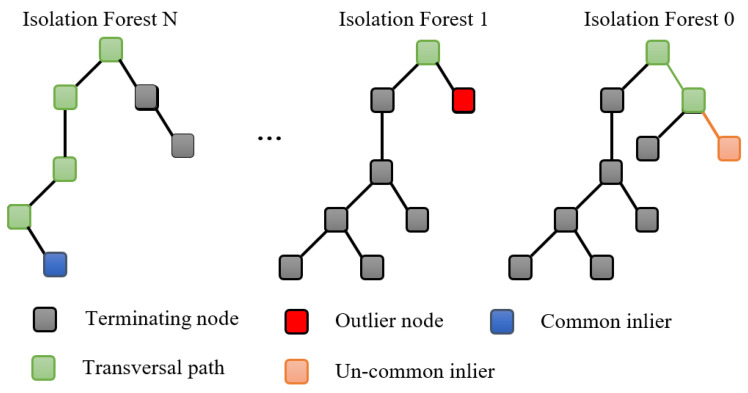
Illustration of anomaly detection using the Isolation Forest technique.

The Isolation Forest is a promising anomaly detection algorithm primarily introduced by Lui in 2008 [13] and enhanced thereafter in 2011 [37]. It is constructed using unlabeled data, making it suitable for practice applications. The principal idea of the IF algorithm consists in identifying anomalies by the isolation of potential outliers from the data [37]. It is inspired based on the Random Forest, which consists of an ensemble of decision trees built in the training step [38]. Visually, Figure 5 depicts the basic construction of the IF, which consists in constructing an ensemble of trees for a given data. Importantly, the iF algorithm recursively divides the data by constructing an ensemble of trees until all samples are separated. Note that anomalies are recognized by a short average path length on the trees [13].

Implementing the iF-based anomaly detection approach demands only two parameters specified: the number of trees and the size of sub-samples used for the splitting operations to build the forest. In [13], it has been demonstrated that the detection performance of the iF approach can converge quickly based on a small number of trees, and it only needs a small sub-sampling size to reach high detection accuracy. In the iF approach, anomalies in a dataset can be detected by analyzing the path lengths for the anomaly data points, with the splitting process being short, which mean that anomalies require few splits in isolation Trees to be isolated [39]. Furthermore, the anomaly score is computed from the mean path length across all the isolation trees in the forest.

Two parameters are to be fixed in implementing the iF algorithm: the number of trees and the size of sub-samples employed for the splitting procedure to construct the forest [13]. Anomalies can be detected using the iF approach by investigating the path lengths for the anomaly data points. Anomalies are distinguished by a short splitting process in isolation Trees to be isolated [39]. The iF algorithm calculates the anomaly score to decide the presence of anomalies based on the mean path length across all the isolation trees in the forest.

Let us consider l(d) the path length of a given data point *d*, and D a dataset constituted of *N* data samples. In the IF approach, log(N) is the minimum depth of a used decision tree, and N−1 is the maximum depth. The anomaly score, A, is computed as follows [13]:

(12) $$A\left(d,N\right)=2^{-\frac{E\left[l(d)\right]}{\alpha (N)}},$$

where $E\left[l(d)\right]$ is the expected path length of a given data point *d* from a collection of isolation trees, and α(N) is the average path length, given as [13]:

(13) $$\alpha \left(N\right)=2\lambda \left(N-1\right)-\frac{2(N-1)}{N},$$

where λ(i) denotes the harmonic number, which could be computed as:

(14) λ(y)=ln(y)+ϵ,

with ϵ denotes the Euler Constant, i.e., ϵ=0.5772156649.

In summary, we obtain the anomaly score of *d*, A(d,N) using iTree from the training data of *N* samples, and the range of A(d,N) is within [0,1]. Note that the anomaly score is oppositely proportional to the path length. Anomaly detection is accomplished, as below.

(15) $$\left\{\begin{array}{cc} \mathrm{an}\;\mathrm{anomaly}\; & \mathrm{if}\;A(d,N)\;\mathrm{is}\;\mathrm{close}\;\mathrm{to}\;1\\ \mathrm{Normal}\;\mathrm{instance} & \mathrm{if}\;A(d,N)\;\mathrm{is}\;\mathrm{close}\;\mathrm{to}\;0\\ \mathrm{Uncertain}\;\mathrm{decision} & \mathrm{if}\;A(d,N)\;\mathrm{is}\;\mathrm{close}\;\mathrm{to}\;0.5 \end{array}\right.$$

## 4. Deep-Learning-Driven Mental Tasks Detector

**Figure 6:**
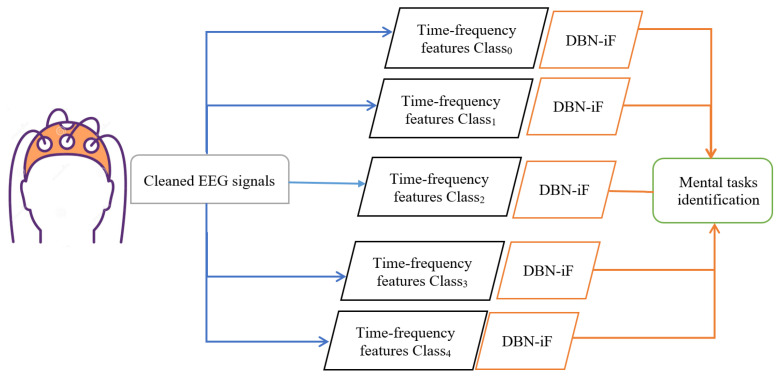
Schematic overview of the proposed DBN-iF approach for mental tasks’ identification using EEG signals.

This study addresses the problem of EEG-based mental tasks classification as multiple anomaly detection channels. Specifically, a hybrid deep generative model is used to model one given class in which its data are regarded as a normal observation, and the data from other classes are abnormal. Here, we first extract time-frequency features of the cleaned EEG data via the QTFD approach. Then, we applied the DBN-based iF detector to identify the mental tasks from the QTFD features of EEG signals, as illustrated in Figure 6. The primary goal for the DBN-iF approach is to identify the mental task from time-frequency features of the EEG signals.

As illustrated in Figure 6, for each class $C_{i}$, we implemented a DBN-based iF approach in an unsupervised manner to identify whether the mental task is from this class or not. Essentially, the *i*th DBN-iF detector is constructed using only the data of the *i*th class, $C_{i}$. Indeed, for a given class $C_{i}$, we trained the DBN in an unsupervised manner using only the data of the $C_{i}$ class is used for the training. This step generates a compact features space for $C_{i}$, which will be used to train a dedicated isolation forest in order to isolate abnormal observations, which are the data points that belong to the other classes, and to keep the observed data points that are normal observation belonging to the current class $C_{i}$. Of course, the 1-vs-all procedure constructs k DBN-iF detectors to separate mental tasks by EEG signals. DBN is composed of a stacked RBM, where each RBM is trained separately in an unsupervised way; this approach is also called greedy layer-wise. This step permits to construct in the hierarchical process a reduced features space containing pertinent information that represents a given class $C_{i}$.

This work’s central idea consists of constructing a DBN-based deep learning model for each EEG signals-based mental task. DBN aims to learn the probability distribution of the underlining training data. DBN is composed of a stacked RBM, where each RBM is trained separately in an unsupervised way; this procedure is also called greedy layer-wise learning. This step permits construction in the hierarchical process a reduced features space containing pertinent information that represents the data of a given class $C_{i}$. The training is performed by estimating the log-likelihood gradient based on the Gibbs Sampling method, Markov Chain Monte Carlo (MCMC) method. The output of unsupervised greedy layer-wise learning is indeed a new compact space constituted of relevant features that effectively represent the mapping of training data points into a latent space L, which is used to feed the iF detector.

The iF approach is applied to the extracted features from the DBN model for EEG-based mental tasks recognition. In other words, the iF scheme is employed to discriminate a given observation as an anomaly if the EGG signal does not belong to target class $C_{i}$ by assigning a high anomaly score (close to one). Otherwise, it assigns a low anomaly score (close to zero) if the EGG signal shares the same features as the target class $C_{i}$. In summary, the DBN-iF is designed for identifying each class separately without any data labeling.

Five statistical scores are computed in this study to compare the studied techniques: Recall, Precision, F1-Score, Accuracy, and Area under curve (AUC) [40]. For binary detection, the number of true positives (TP), false positives (FP), false negatives (FN), and true negatives (TN) are used to calculate the statistical scores.

(16) $$\mathrm{Accuracy}=\frac{\mathrm{TP}+\mathrm{TN}}{\mathrm{TP}+\mathrm{FP}+\mathrm{TN}+\mathrm{FN}}.$$

(17) $$\mathrm{Recall}=\frac{\mathrm{TP}}{\mathrm{TP}+\mathrm{FN}}.$$

(18) $$\mathrm{Precision}=\frac{\mathrm{TP}}{\mathrm{TP}+\mathrm{FP}}.$$

(19) $$F1-\mathrm{Score}=2\frac{\mathrm{Precision}.\mathrm{Recall}}{\mathrm{Precision}+\mathrm{Recall}}=\frac{2\mathrm{TP}}{2\mathrm{TP}+\mathrm{FP}+\mathrm{FN}}.$$

## 5. Results and Discussion

### 5.1. Data Description

This part is dedicated to evaluating the efficacy of the presented technique in recognizing different classes; experiments are conducted via actual data from the Institute for Knowledge Discovery. The EEG signals are recorded by nine different patients with disabilities (spinal cord injury and stroke) on distinct sessions. Nine patients completed a specific experimental paradigm, including five mental tasks (MT): mental word association (condition WORD), spatial navigation (NAV), mental subtraction (SUB), feet motor imagery (FEET), and right-hand motor imagery (HAND. See [41] for more details.

**Figure 7:**
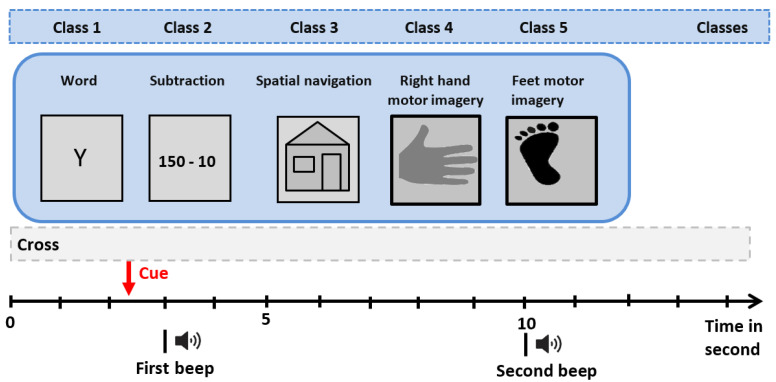
Description of data acquisition procedure.

The experiment protocol was carried out over several days, where for each day, a single subject session (involved eight runs resulting in 40 trials) is recorded. Each experimental run contained 25 cues with five different mental tasks. Cues (indicating different classes) were put in random ranking to allow a fair evaluation. EEG signals were then acquired from 30 electrodes positioned on the scalp according to the international 10–20 protocol. This protocol was developed to maintain standardized testing methods ensuring that a patient’s study outcomes could be reproduced and effectively analyzed and compared to previously obtained results in the literature [42]. The locations of electrode contained channels AFz, F7, F3, Fz, F4, F8, FC3, FCz, FC4, T3, C3, Cz, C4, T4, CP3, CPz, CP4, P7, P5, P3, P1, Pz, P2, P4, P6, P8, PO3, PO4, O1, and O2. Channels corresponding to reference and ground have been placed at the left and right mastoid. For better representativeness, an overview of the experimental protocol is given in Figure 7. The acquisition was carried out using the g.tec GAMMAsys system, g.USBamp biosignal amplifiers and g.LADYbird active electrodes (Guger Technologies, Graz, Austria).

After the acquisition phase, several processing operations were applied to the EEG signals: bandpass filter 0.5–100 Hz (notch filter at 50 Hz) and sampling stage at a rate of 256 Hz. The period of a single imagery experiment consists of ten seconds. In the beginning, a cross is shown on the screen, and participants are requested to relax and fixate the cross in order to avoid eye motions. A beep is given after three seconds to bring the participant’s attention. The cue revealing the asked imagery task, one out of five graphical symbols, was shown between the time interval t = 3 s and t = 4.25 s. Then, a second beep was conducted at time t = 10 s, and the fixation-cross was removed, indicating the trial’s end. Before the subsequent trial, a variable break (intertrial interval, ITI) stayed around 2.5 s and 3.5 s. Participants are requested to escape moving during the imagery period and avoid moving and blinking within the ITI. A blank screen is displayed for four seconds in the beginning and end of the experiment. For more details about this data, refer to [41].

### 5.2. Experiments and Settings

The proposed approach aims to build a discriminate method able to distinguish and classify EEG signal of a give motion. This section is dedicated to assessing the performance of the proposed detector in discriminating five distinct mental tasks based on EEG signals, namely mental word association, mental subtraction, spatial navigation, right-hand motor imagery, and feet motor imagery. After removing artifacts from EEG signals using the MWF procedure, QTFD is applied to generate a high-resolution time-frequency representation of the EEG signals and catch the EEG signals’ spectral variations over time. After that, the extracted QTFD features are used as input to the proposed approach for EEG signals classification. The study aims to build an unsupervised discriminate method able to distinguish and classify EEG signals of a given motion—specifically; there are five classes (five distinct mental tasks), namely mental word association, mental subtraction, spatial navigation, right-hand motor imagery, and feet motor imagery. The proposed approach addresses the problem of classification as a multi anomaly detection problem.

The dataset used consists of five classes, and we create a sub-set composed of only data points of a given class (target). Further, the training dataset is composed of 80%, a sub-set of the target class, and 20% will be used for the testing. Moreover, we create a testing data set composed of two parts 20% of the remaining data of the target class (as a normal observation: inliers), and we selected randomly 20% from the other classes (as abnormal or anomalies: outliers) to construct a testing dataset containing an amount of data of all classes. We repeat this procedure (testing) for all classes. This paper integrates a DBN model for feature extraction and an IF approach to recognize a given EEG signal class. In other words, we design a DBN-based IF detector for each class. This study is conducted using an ordinary PC with CPU i7 and 12Go RAM based on Ubuntu 20. The investigated methods are implemented using Python. Specifically, TensorFlow 2.3 and Keras 2.3 are used to implement the DBN, and Scikit-learn 1.1. to perform the detection via the Isolation Forest algorithm.

In the training phase, we fine-tune the parameters of the proposed approach using the training data via the grid search procedure. Specifically, the DBN model training employs a greedy layer-wise training procedure. The selected DBN model comprises three layers (30, 15, 5) hidden units, i.e., two stacked RBMs. Here, we adopted the following hyper-parameters: the number of Gibbs steps performed is 5, the learning rate used is 0.001, the number of epochs during the training is 1000 with a batch size of 250. Essentially, we train each RBM first in an unsupervised way, and then the IF detector will be applied to the output of the DBN model. The values of IF parameters are chosen in training so that the number of false alarms is reduced. The IF model constructs multiple isolation trees; in our study, we used N=150 the number of trees and the size of the sub-samples 256. We compare the computed iF-driven anomaly score to a detection threshold of 0.5. If the calculated anomaly score overpass 0.5, it is a confirmed anomaly; otherwise, it is considered a normal observation. However, a special case may occur when the anomaly score is equal to 0.5 or close to 0.5, and this situation is considered an uncommon normal observation.

In this study, we considered two other commonly used anomaly detection schemes, namely Elliptical Envelope (EE) [43], and Local Outlier Factor (LOF) [14]. Specifically, we compare the performance of the proposed DBN-based IF approach with that of DBN-based DCF and LOF methods. Similar to the DBN-IF, in DBN-EE and DBN-LOF, the EE and LOF detectors are applied to the extracted features from DBN to distinguish and classify the EEG signal of a given motion. We construct a model using training data based on unsupervised learning for each EEG signal class. In the LOF approach, the anomaly score is calculated for each data point by calculating the local divergence of the density of a given sample in comparison to its neighbors. Here, the number of neighbors in LOF is 35. On the other hand, the EE approach fits an ellipse around the data utilizing a minimum covariance determinant (MCD). In this experiment, the proportion of points to be enclosed in support of the raw MCD estimation is 0.9.

### 5.3. Discussion and Analysis

Now, we applied the trained DBN-iF model to the testing data to identify five distinct mental tasks based on EEG signals. As discussed above, we addressed this multiclass classification problem as multiple binary anomaly detection problems. Note that features characterizing the other classes are viewed as anomalies or outliers by the DBN-iF detector, and features from the targeted class are considered normal and should not be flagged as an outlier during the testing phase. Indeed, a single DBN-iF detector is constructed based on training data of each class, with the samples of that class as inlier and all other samples as anomalies (i.e., one-vs.-rest). Detection results of the DBN-iF are summarized in Table 1. The column *class* in Table 1 represents the target class, meaning that the training data contains only the data of this class, while the column *Others* denotes the remaining classes where their data are used for the testing to evaluate the performance of the instances of the hybrid model used fro the target class. Table 1 reveals the high capacity of the DBN-iF in identifying data from the first class (WORD) from other classes (SUB, NAV, HAND, and FEET) by obtaining a high ACU values of $AUC_{2}=0.9779$, $AUC_{3}=0.9870$, $AUC_{4}=0.9902$, and $AUC_{5}=0.9840$. This means that the DBN-iF detector can efficiently recognize the first task from other tasks in other classes. Furthermore, results in Table 1 show that class 2 detection performance is very high, where the average AUC is 0.9918 for all classes. The averaged AUC values achieved by the DBN-iF detector for the third, fourth, and fifth classes are 0.9895, 0.9685, and 0.99, respectively. It can be observed from this first experiment that the DBN-iF approach provided satisfactory identification of mental tasks by EEG signals. The obtained high performance demonstrates the efficiency of the amalgamation of the deep learning model, DBN, with the Isolation Forest detector in recognizing mental tasks (e.g., motor imagery, calculation, reading) based on EEG signals. This could be attributed to different factors, including (i) the extended capacity of the QTFD in extracting spectral variations of the EEG signals, (ii) the flexibility of robustness of the DBN to approximate the data distribution of the underlining EEG signal class through creating a latent space that represents much better the original data (EEG signal) separately, and (iii) the sensitivity of the iF scheme in detecting anomalies through branching paths. In addition, this approach focuses on each class separately, making it easy to be separated from other classes.

This study compared three different outlier detection models for mental task identification by EEG signals: DBN-based iF, LOF, and EE schemes. Results of DBN-based LOF and EE schemes are listed in Table 2 and Table 3. From Table 2, we observe that the DBN-LOF scheme recognized classes 3 and 5 with high accuracy with an average AUC score of 0.9689 and 0.9768, respectively. However, it achieved relatively moderate identification of metal tasks related to classes: 1, 2, and 4 with the average AUC of 0.9208, 0.9382, and 0.9120, respectively. Results in Table 3 indicate that EE performance was high for classes: 3 and 5 with an AUC average great than 0.95, while it is lower than 0.9 with 0.8908, 0.8889, and 0.8429 for classes 1, 2, and 4, respectively. Of course, from Table 2 and Table 3 we can assume that DBN-LOF scheme outperformed the DBN-EE scheme.

**Figure 8:**
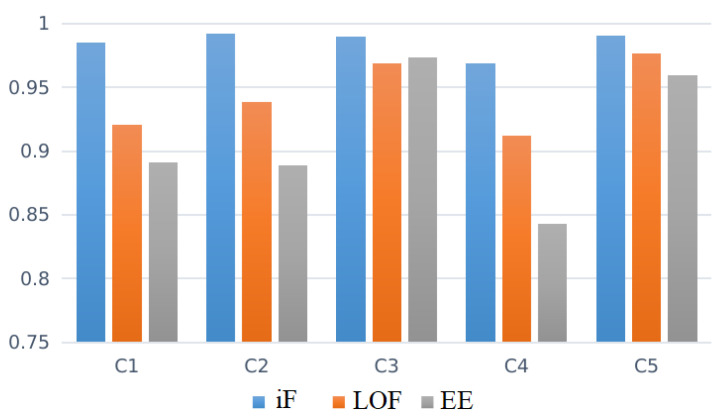
AUC obtained per detector for each EEG signal class.

**Figure 9:**
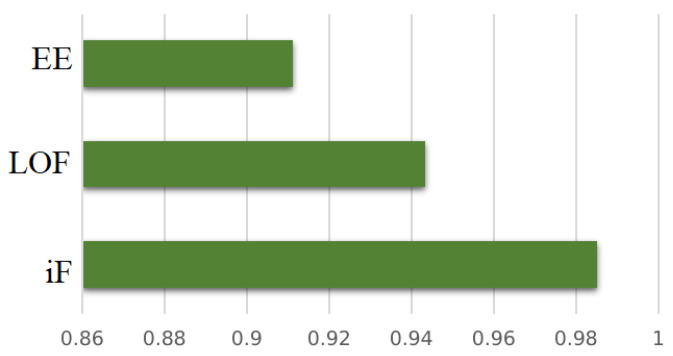
Averaged AUC obtained per anomaly detector for all EEG signal classes.

Table 4 shows the aggregated AUC obtained by three investigated approaches: DBN-iF, DBN-LOF, and DBN-EE. Figure 8 displays the barplot of averaged AUC to visually aid the comparison of achieved results by the three considered schemes. Figure 9 illustrates the aggregated results of those shown in Table 4.

It would appear, based on results in Table 4 Figure 8 that DBN-iF would be the best model for mental task recognition based on EEG signals. It dominates the other models (DBN-LOF and DBN-EE) by better identifying mental tasks from the acquired EEG signals.

### 5.4. Comparison with the State-of-the-Art

Lastly, to measure the real contribution of the present work, comparisons with some existing and recent systems conducted on EEG classification datasets are reported in Table 5. Several powerful classifiers are invoked, namely: least squares support vector machine [3,6], convolutional neural networks and stacked autoencoders [23], KNN [8], logistic regression [11], and LR KLR, MLP, PNN, and LS-SVM [7]. From Table 5, the results demonstrate the outperformance of the proposed approach over classifying EEG signals by the state-of-the-art approaches, even in critical scenarios. The proposed approach dominates the other methods mainly because of its capability to recognize each class individually and approximate the data distribution by the generation of latent space, which is suitable in the representation of original data.

In the case of the logistic regression formalism [11] (applied alone or kernalised LR), one can observe some misclassification cases, which impacted the recognition rate (lower than 95%). In [23], a CNN-SAE combination has been used. One can mention the presence of numerous misclassification cases, which affects the accuracy presenting the lowest classification accuracy (90%). This could be explained by CNN formalism being more adapted for image or matrix classification (data in 2D form) than signal classification (EEG samples). Importantly, transforming EEG signals from 1D to 2D to obtain matrix representation can cause the loss of some pertinent information. In [3,6,7,11], an LS-SVM combination formalism has been used as a recognition algorithm, which achieved a relatively high accuracy of around 96%. Even if SVMs formalism is based on geometric aspect, where the samples’ separation is established on the sparse solution via structural risk minimization, the DBN classification has outperformed the LS-SVM combinations. In summary, all of these observations confirm that the DBN formalism is more adapted to EEG signals’ classification than several existing methods.

## 6. Conclusions

This study introduced an unsupervised deep learning-based strategy for discriminating the mental tasks of EEG signals. Essentially, multiclass classification is handled as an anomaly detection problem without using labeled data (i.e., fully unsupervised). After removing artifacts from EEG signals, a time-frequency representation of the EEG signals is obtained using the QTFD and used as input of the designed detector to improve mental tasks’ recognition. The iF anomaly detection scheme is applied to the features extracted by the DBN model for separating mental tasks from the EEG signals. This approach is assessed on publicly available benchmark EEG datasets comprising five classes. We compared the DBN-iF approach with two other unsupervised detection approaches, DBN-based EE and LOF. Results demonstrated that the DBN-iF delivers superior discrimination performance of mental tasks by EEG Signals and dominates the investigated methods, DBN-CDE and DBN-LOF. It has demonstrated that merging the DBN deep learning model with anomaly detection methods presents a promising strategy to mitigate challenges in EEG-based mental tasks’ recognition.

Despite the encouraging results for MI-based tasks recognition obtained using the EEG-based mental tasks recognition via a deep learning-driven anomaly detector approach, this work raises some directions of improvement that merit consideration from researchers for future work. In particular, the extended recognition of EEG signals is to perform tasks and establish possible communication between several patients via BCI applications.

In terms of methodology, this study demonstrated that the proposed DBN-driven Isolation Forest approach achieved satisfactory discrimination results. However, the DBN model does not consider feature selection, and the considerable amount of irrelevant data in high-dimensional data can constrain its performance. Therefore, we plan to develop an improved DBN model that focuses only on relevant features by integrating attention mechanisms within the DBN model; the attention mechanism allows the model to focus on essential features [46].

On the other hand, the Isolation Forest algorithm is computationally efficient and has proven effective in anomaly detection. However, its final anomaly score depends on the contamination parameter provided during the training stage. This implies that we need to have an idea of what percentage of the training data is anomalous beforehand to obtain a better prediction. In addition, it has the disadvantage of sensing local anomaly points, which influences the algorithm’s precision [47]. As an alternative, we plan in future work to develop deep model-driven statistical monitoring schemes by merging the capacity of generative deep learning models, such as generative adversarial networks (GANs) [48] and variational autoencoders (VAE) [49], to find low-dimensional summaries that will be monitored by statistical monitoring charts, such as the generalized likelihood ratio (GLR) test [50]. 

## Figures and Tables

**Table 1:** DBN-driven iF identification results.

CLASS	OTHERS	Accuracy	Precision	F1-Score	AUC
1	2	0.9779	0.9666	0.9781	0.9779
1	3	0.9870	0.9841	0.9870	0.9870
1	4	0.9902	0.9905	0.9902	0.9902
1	5	0.9840	0.9782	0.9840	0.9840
2	1	0.9918	0.9936	0.9918	0.9918
2	3	0.9912	0.9925	0.9912	0.9912
2	4	0.9924	0.9948	0.9924	0.9924
2	5	0.9920	0.9941	0.9920	0.9920
3	1	0.9917	0.9934	0.9917	0.9917
3	2	0.9867	0.9836	0.9867	0.9867
3	4	0.9909	0.9918	0.9908	0.9909
3	5	0.9892	0.9883	0.9892	0.9892
4	1	0.9806	0.9716	0.9808	0.9806
4	2	0.9593	0.9327	0.9605	0.9593
4	3	0.9762	0.9636	0.9766	0.9762
4	5	0.9580	0.9304	0.9593	0.9580
5	1	0.9910	0.9921	0.9910	0.9910
5	2	0.9881	0.9862	0.9881	0.9881
5	3	0.9899	0.9898	0.9899	0.9899
5	4	0.9917	0.9934	0.9917	0.9917

**Table 2:** DBN-driven LOF identification results.

CLASS	OTHERS	Accuracy	Precision	F1-Score	AUC
1	2	0.9083	0.8498	0.9154	0.9083
1	3	0.9172	0.8628	0.9229	0.9172
1	4	0.9779	0.9647	0.9782	0.9779
1	5	0.8799	0.8103	0.8920	0.8799
2	1	0.8983	0.8345	0.9072	0.8983
2	3	0.9321	0.8847	0.9360	0.9321
2	4	0.9621	0.9346	0.9632	0.9621
2	5	0.9604	0.9317	0.9617	0.9604
3	1	0.9607	0.9313	0.9620	0.9607
3	2	0.9588	0.9279	0.9602	0.9588
3	4	0.9951	0.9955	0.9951	0.9951
3	5	0.9611	0.9320	0.9623	0.9611
4	1	0.9050	0.8463	0.9124	0.9050
4	2	0.8979	0.8363	0.9065	0.8979
4	3	0.9383	0.8975	0.9413	0.9383
4	5	0.9070	0.8492	0.9141	0.9070
5	1	0.9478	0.9107	0.9501	0.9478
5	2	0.9866	0.9805	0.9867	0.9866
5	3	0.9850	0.9774	0.9851	0.9850
5	4	0.9876	0.9823	0.9876	0.9876

**Table 3:** DBN-driven EE identification results.

CLASS	OTHERS	Accuracy	Precision	F1-Score	AUC
1	2	0.9187	0.8664	0.9241	0.9187
1	3	0.8995	0.8383	0.9078	0.8995
1	4	0.9015	0.8411	0.9095	0.9015
1	5	0.8436	0.7659	0.8636	0.8436
2	1	0.7846	0.7017	0.8213	0.7846
2	3	0.9122	0.8567	0.9185	0.9122
2	4	0.9421	0.9035	0.9447	0.9421
2	5	0.9168	0.8637	0.9225	0.9168
3	1	0.9606	0.9350	0.9617	0.9606
3	2	0.9716	0.9550	0.9722	0.9716
3	4	0.9905	0.9912	0.9905	0.9905
3	5	0.9718	0.9553	0.9723	0.9718
4	1	0.9172	0.8643	0.9228	0.9172
4	2	0.7579	0.6762	0.8035	0.7579
4	3	0.9134	0.8585	0.9195	0.9134
4	5	0.7833	0.7004	0.8204	0.7832
5	1	0.9126	0.8574	0.9189	0.9126
5	2	0.9769	0.9648	0.9772	0.9769
5	3	0.9790	0.9687	0.9792	0.9790
5	4	0.9705	0.9528	0.9710	0.9705

**Table 4:** Overall detection results of all anomaly detection methods.

CLASS	iF	LOF	EE
1	0.9848	0.9208	0.8908
2	0.9919	0.9382	0.8889
3	0.9896	0.9689	0.9736
4	0.9685	0.9120	0.8429
5	0.9902	0.9768	0.9597

**Table 5:** Comparison of the proposed method with existing methods.

Paper	The Used Features	Approach	Accuracy (%)
[6]	EMD	LS-SVM	97.56
[3]	TQWT	LS-SVM	96.89
[23]	STFT and electrode location information	CNN-SAE	90
[8]	MSPCA, DWT and WPD	KNN	92.8
[11]	cross-correlation and DWT coefficients	LR	92.3
		KLR	94.3
		MLP	94.9
		PNN	92.9
		LS-SVM	96.1
[7]	Optimal allocation features	LS-SVM	96.62
		Naive Bayes	96.36
[44]	EEG-inception (time-series signals) with data augmentation	CNN	88.58
[45]	Semantic, intrinsic, and user-specific features (with data augmentation)	multi-scale CNN	93.74
**This study**	QTFD	DBN-iF	98.5

## Data Availability

Not applicable.
